# Production of blastocysts following in vitro maturation and fertilization of dromedary camel oocytes vitrified at the germinal vesicle stage

**DOI:** 10.1371/journal.pone.0194602

**Published:** 2018-03-15

**Authors:** Mohamed Fathi, Adel R. Moawad, Magdy R. Badr

**Affiliations:** 1 Department of Theriogenology, Faculty of Veterinary Medicine, Cairo University, Giza, Egypt; 2 Department of Artificial Insemination and Embryo Transfer, Animal Reproduction Research Institute, Agriculture Research Centre, Giza, Egypt; Institute of Zoology Chinese Academy of Sciences, CHINA

## Abstract

Cryopreservation of oocytes would serve as an alternative to overcome the limited availability of dromedary camel oocytes and facilitate improvements in IVP techniques in this species. Our goal was to develop a protocol for the vitrification of camel oocytes at the germinal vesicle (GV) stage using different cryoprotectant combinations: 20% EG and 20% DMSO (VS1), 25% EG plus 25% DMSO (VS2) or 25% EG and 25% glycerol (VS3) and various cryo-carriers; straws or open pulled-straw (OPS) or solid surface vitrification (SSV); and Cryotop. Viable oocytes were cultured in vitro for 30 h. Matured oocytes were fertilized with epididymal spermatozoa and then cultured in vitro in modified KSOMaa medium for 7 days. Survival and nuclear maturation rates were significantly lower (*P ≤* 0.05) in oocytes exposed to VS3 (44.8% and 34.0%, respectively) than those exposed to VS1 (68.2% and 48.0%, respectively) and VS2 (79.3% and 56.9%, respectively). Although recovery rates were significantly lower (*P ≤* 0.05) in SSV and Cryotop vitrified oocytes (66.9% to 71.1%) than those vitrified by straws with VS1 or VS2 solutions (86.3% to 91.0%), survival rates were higher in the SSV and Cryotop groups (90.7% to 94.8%) than in the straw and OPS groups (68.2% to 86.5%). Among vitrified groups, maturation and fertilization rates were the highest in the Cryotop-VS2 group (51.8% and 39.2%, respectively). These values were comparable to those seen in the controls (59.2% and 44.6%, respectively). Cleavage (22.5% to 27.9%), morula (13.2% to 14.5%), and blastocyst (6.4% to 8.5%) rates were significantly higher (*P ≤* 0.05) in SSV and Cryotop groups than in straws. No significant differences were observed in these parameters between the Cryotop and control groups. We report for the first time that dromedary oocytes vitrified at the GV-stage have the ability to be matured, fertilized and subsequently develop in vitro to produce blastocysts at frequencies comparable to those obtained using fresh oocytes.

## Introduction

Oocyte cryopreservation has potential applications in human and animal reproduction, such as fertility preservation in cancer patients, preservation of genetic materials in livestock and conservation of endangered species [1, 2]. However, compared with the reported successes in the fields of sperm and embryo cryopreservation, the outcomes of oocytes cryopreservation remain unsatisfactory [3]. Although two primary methods, slow-rate freezing and vitrification, have been developed to preserve oocytes, vitrification is favored because it produces less damage [4]. To accomplish proper vitrification, high cooling rates in combination with high concentrations of cryoprotectants should be considered. Various techniques, such as Cryotop [5], cryoloop [6], solid surface vitrification (SSV) [7], nylon mesh [8], cryoleaf [9] and open pulled straw (OPS) [10], have been developed to achieve rapid cooling by reducing the volume of the vitrification solutions. Exposure of oocytes to high concentrations of cryoprotectants during vitrification is toxic and can cause zona hardening and parthenogenetic activation, which negatively impact the fertilization ability and development of vitrified/warmed oocytes [11]. These toxic effects could be minimized by several approaches including pretreatment of oocytes with lower concentrations of cryoprotectants before exposure to the final vitrification solutions, controlling the time of exposure and selecting the least toxic permeating cryoprotectant agents [12]. Various permeating cryoprotectants such as ethylene glycol (EG), dimethyl sulfoxide (DMSO), glycerol, propylene glycol, 1,2-propanediol (PROH) and glycerol have been successfully used for the vitrification of oocytes and embryos in different mammalian species including camels [13–26]. Although extensive research has been conducted on the cryopreservation of metaphase II (MII) oocytes, vitrification of oocytes at this stage can disrupt the meiotic spindle [27], which could be avoided by freezing the oocytes at the germinal vesicle (GV) stages. Cryopreservation of GV-oocytes has been reported in various species [15–21, 24, 28–30], and successful production of live offspring from frozen/thawed GV-oocytes has been demonstrated in humans, mice, and cattle [31–33]; however, no comparable studies have been performed in dromedary camels.

The dromedary camel, *Camelus dromedarius*, is an important livestock species with high productivity of meat and milk. However, the reproductive efficiency in camelids is low, partly due to the late onset of puberty, early embryonic mortality, seasonality and the length of the gestation period (13 months). Although recent reproductive technologies such as IVF [34, 35] and somatic cell nuclear transfer (SCNT) [36] have been successfully applied to camelids and the birth of live offspring following these technologies has been reported [36, 37], in vitro embryo production (IVP) is still not well-developed in this species compared with other domestic species. One of the main factors contributing to this situation is the limited availability of oocytes due to the shortage of abattoirs slaughtering female camels as well as the lack of a standardized protocol for in vitro maturation (IVM) and IVF of dromedary camel oocytes. Cryopreservation of oocytes would be an alternative way to overcome the limited availability of oocytes, allowing for improvements in IVP in this species. Therefore, the objective of the present study was to develop a protocol for the vitrification of dromedary camel cumulus oocyte complexes (COCs) at the GV-stage using various cryoprotectant combinations and cryo-carriers. First, we evaluated the viability and nuclear maturation of dromedary camel GV-oocytes after exposure to different vitrification solutions: VS1 (20% EG plus 20% DMSO); VS2 (25% EG plus 25% DMSO); and VS3 (25% EG plus 25% glycerol). Then, we compared in vitro maturation, fertilization and preimplantation embryo developmental rates of dromedary camel oocytes vitrified at GV stages using different cryo-carriers (straws, OPS, SSV and Cryotop) and different cryoprotectants (VS1 and VS2).

## Materials and methods

Unless stated otherwise, all chemicals and reagents were purchased from Sigma–Aldrich (St Louis, MO, USA).

### Collection of dromedary camel cumulus-oocyte complexes

Dromedary camel ovaries were collected from a local slaughterhouse (Cairo, Egypt) over the period of November 2016 to April 2017 and kept in a thermos flask filled with pre-warmed (30 °C), sterile, normal saline solution (NSS, 0.9% NaCl) until processing. COCs were aspirated from 2 to 8 mm follicles using a 20-gauge needle attached to a 20-mL syringe [34, 35]. The follicular fluid containing the COCs was placed into 50-mL conical tubes with washing medium (HEPES buffered-TCM 199 (H-TCM 199) supplemented with 10% (vol/vol) fetal calf serum (FCS)), and maintained at 39 °C for 10 minutes, causing the COCs to settle to the bottom of the tubes. The follicular fluid containing the COCs was poured into a 100-mm Petri dish. COCs with at least one to three layers of compact cumulus cells and a homogenous ooplasm were selected, using stereomicroscopy, for further experiments. All experiments were approved by Institutional Animal Care and Use Committee (IACUC), Cairo University, Egypt.

### Toxicity test

To evaluate the effects of exposure of COCs to different vitrification solutions (VS) on survival and nuclear maturation, selected COCs were equilibrated in a solution of 10% EG and 0.25 M trehalose (ES) for 3 min at 37 °C. Following equilibration, COCs were exposed for 60 sec to VS1 (20% EG plus 20% DMSO), VS2 (25% EG plus 25% DMSO) or VS3 (25% EG plus 25% glycerol). After exposure, dilution of the cryoprotectants was performed by transferring the COCs into 1 M trehalose solution, where they were maintained for 3 min at 37 °C. COCs were then transferred into decreasing concentrations of trehalose solution (0.5 and 0.25 M) for 3 min each at room temperature. All vitrification and dilution solutions were prepared in H-TCM 199 medium supplemented with 10% (v/v) fetal calf serum (FCS) as a base medium (BM). Following cryoprotectant dilution, COCs were morphologically examined according to a previously described method [19].

### Vitrification and warming of COCs

After equilibration in ES as mentioned above, COCs were transferred to either VS1 or VS2 in BM, at room temperature, for 60 sec before being loaded into the cryodevice (straws, OPS, Cryotop, or SSV). Vitrification of oocytes by straws, OPS or SSV was performed according to the methods previously described [7, 10, 24]. Vitrification with Cryotop was performed according to the method described by [5] as follows. Groups of 5 COCs that had been exposed to either VS1 or VS2 were loaded on thin polypropylene strips of Cryotop and directly plunged into LN_2_. Then, the thin strip was covered with a hard plastic cover (3 cm long) on top of the Cryotop sheet to protect it during storage in the liquid nitrogen containers. Warming was performed according to the method described by [24] with minor modifications. Briefly, vitrified oocytes were transferred to 1 M trehalose solution in BM at 37 °C for 3 min; then, they were placed into a BM supplemented with 0.5 and 0.25 M trehalose and then in BM for 3 min each at room temperature. Finally, intact COCs were determined morphologically according to the method described previously [19]

### Oocyte IVM, evaluation of cumulus expansion and nuclear maturation

IVM of COCs was performed as previously described [34, 35]. Briefly, after washing twice in washing medium and once in maturation medium (TCM-199 with Earle’s salts, supplemented with 10 μg/mL oFSH, 10% FCS, 50 μg/mL sodium pyruvate, 2.6 mg/mL sodium bicarbonate, and 50 μg/mL gentamycin), groups of 10 to 15 COCs were cultured in 100 μL of pre-warmed maturation medium under mineral oil for 30 h at 39 °C in 5% CO2 in air. After IVM for 30 h, the proportions of COCs with expanded, and loosened cumulus cell layers were identified using stereomicroscopy. Nuclear maturation was assessed using aceto-orcein staining according to the method described by [35, 38]. In brief, denuded oocytes were incubated in 1% hypotonic sodium citrate solution for 3 minutes. Oocytes were then placed on a glass slide under a coverslip. The slides were fixed in a solution of ethanol:acetic acid (3:1) for at least 24 h. After fixation, the oocytes were stained with 1% orcein, and the slides were then examined using phase contrast microscopy. Based on the chromatin configuration, oocytes at the MII stage were recorded as mature.

### IVF and evaluation of fertilization status

Mature oocytes were fertilized in vitro using epididymal spermatozoa according to the method described previously [34, 35, 38]. Briefly, testicles collected from 6 mature male dromedary camels were transported to the laboratory in NSS at 30 °C. The testes were washed twice with NSS, and spermatozoa were collected from the epididymides by a flushing technique. A small incision was made in the body of the epididymis using a sterile sharp scalpel. A 20-gauge sterile needle attached to a 5 mL syringe, filled with flushing medium (Sperm-TALP medium), was inserted into the incision. The flushing medium was gently pushed toward the cauda epididymides, and a slight digital pressure was applied all over the epididymis. Another small incision was made in the cauda epididymides. Droplets of the flushing medium containing the spermatozoa were collected in a 100-mm Petri dish. The medium containing the spermatozoa was kept at 39 °C under 5% CO_2_ in air for 10 minutes before being transferred to a 15-mL centrifuge tube. After centrifugation and removal of the supernatant, the sperm pellet was re-suspended in 1 mL of sperm-TALP medium supplemented with 5 mM caffeine. Following swimming for 1 hour at 39 °C, sperm motility was evaluated. For IVF, in vitro mature oocytes were washed three times in fertilization medium (TALP supplemented with 6 mg/mL BSA, 50 μg/mL gentamycin, and 5 mM caffeine) and then inseminated with motile spermatozoa at concentration of 2 x 10^6^ spermatozoa/mL. Oocytes and spermatozoa were co-incubated for 18 h at 39 °C in 5% CO2 air. Following gamete co-incubation, the fertilization status was determined by fixation and staining of oocytes using aceto-orcein staining as described above. Inseminated oocytes were categorized as fertilized when a spermatozoon, swollen sperm head, or a male and/or female pronucleus was detected in the ooplasm.

### In vitro culture and embryo evaluation

Eighteen hours post-insemination (pi), presumptive zygotes were washed three times in H-TCM 199 supplemented with 5% FCS and then twice in embryo culture medium (modified KSOMaa [35, 39]) and cultured in groups of five zygotes per 50 μL drops of embryo culture medium under mineral oil at 39 °C in a humidified atmosphere of 5% CO_2_, 5% O_2_, and 90% N_2_ until Day 7 (Day 0 = day of insemination). Cleavage, morula and blastocyst development were recorded on Days 2, 4 and 7 pi, respectively.

### Statistical analysis

At least three replicates were used for each experimental group. Data were presented as the means ± S.E.M. and analyzed by one-way ANOVA followed by Tukey's multiple comparisons test. Student’s *t*-test was used to evaluate the differences in blastocyst development among the experimental groups. Statistical analysis was performed using GraphPad Prism 5 software. The results were considered to be statistically significant at *P ≤* 0.05.

## Results

### Effects of exposure of dromedary camel COCs to different combinations of cryoprotectants on survival and nuclear maturation

**Fig 1:**
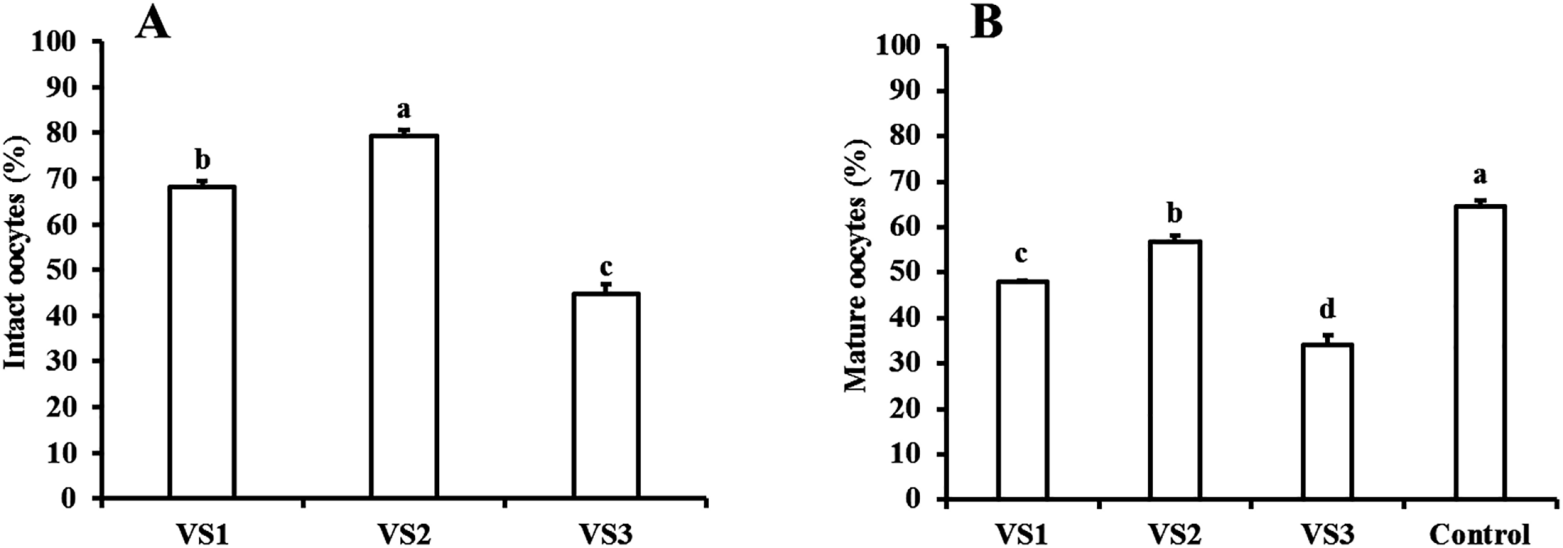
Survival (based on morphological evaluation) and nuclear maturation of dromedary camel GV-oocytes after exposure to different combinations of cryoprotectants. (A) Percentages of morphologically normal oocytes in dromedary camel COCs exposed to various vitrification solutions; VS1 (20% EG plus 20% DMSO), VS2 (25% EG plus 25% DMSO) or VS3 (25% EG plus 25% glycerol); (B) Percentages of in vitro matured oocytes (oocytes at MII-stage) after IVM of dromedary camel GV-oocytes exposed to different vitrification solutions. Data are presented as the means ± S.E.M. Different small letters indicate significant differences at *P ≤* 0.05.

Exposure of COCs to VS3 significantly (*P ≤* 0.05) reduced the percentages of morphologically intact oocytes compared to those exposed to VS1 or VS2 (44.8%, 68.2% and 79.3%, respectively) (Fig 1A). Furthermore, there was a significant difference between VS1 and VS2 in terms of oocyte survival. Following IVM, the percentages of oocytes reaching the MII-stage were significantly (*P ≤* 0.05) lower in the VS3 group compared with those in the VS1 and VS2 groups (34.0%, 48.0% and 56.9%, respectively). These values were significantly (*P ≤* 0.05) lower than those seen in the controls (64.5%) (Fig 1B).

### Recovery rates and survival of dromedary camel oocytes vitrified at the GV-stage using different cryo-carriers and various vitrification solutions

Percentages of recovered oocytes following vitrification and warming were significantly (*P ≤* 0.05) lower in the Cryotop vitrified groups (62.6% and 66.9% in VS1 and VS2, respectively) compared with those vitrified in straws (86.3% and 91.0% in VS1 and VS2, respectively) and with those vitrified in OPS (79.6% and 84.9% in VS1 and VS2, respectively). The recovery rates were also reduced in SSV vitrified groups (69.0% and 71.1% in VS1 and VS2, respectively) compared with those vitrified in traditional-straws (Table 1). The highest proportions (*P ≤* 0.05) of morphologically intact oocytes were found in the SSV and Cryotop groups, with the values ranging from 90.7% to 94.8%, compared to those obtained after vitrification and warming using traditional straws (68.2% and 70.9% in VS1 and VS2, respectively) or to those obtained after OPS vitrification using the VS1 solution (78.7%, Table 1).

**Table 1:** Recovery rates and survival of dromedary camel oocytes vitrified at the germinal vesicle stage using different cryoprotectant combinations and various cryodevices.

Cryodevice	Cryoprotectant	Oocytes	Recovered oocytes n (mean% ± SEM)	Intact oocytes n (mean% ± SEM)*
**Straw**	VS1	136	117 (86.3 ± 1.9)^ac^	80 (68.2 ± 2.3)^d^
	VS2	132	120 (91.0 ± 0.7)^c^	85 (70.9 ± 0.7)^d^
**OPS**	VS1	118	94 (79.6 ± 0.7)^ad^	74 (78.7 ± 0.6)^c^
	VS2	121	103 (84.9 ± 2.2)^ac^	89 (86.5 ± 1.0)^b^
**SSV**	VS1	125	86 (69.0 ± 2.1)^be^	78 (90.7 ± 0.9)^ab^
	VS2	130	92 (71.1 ± 2.7)^bd^	87 (94.6 ± 1.0)^a^
**Cryotop**	VS1	110	69 (62.6 ± 1.4)^e^	64 (92.8 ± 1.3)^a^
	VS2	115	77 (66.9 ± 1.1)^be^	73 (94.8 ± 1.3)^a^

VS1—oocytes vitrified in a vitrification solution composed of 20% EG plus 20% DMSO.

VS2—oocytes vitrified in a vitrification solution composed of 25% EG plus 25% DMSO.

* indicates that viability was calculated as the number of morphologically normal oocytes over the number of recovered oocytes after vitrification and warming.

Values with different superscripts in the same column are significantly different at *P ≤* 0.05.

### Cumulus cell expansion and nuclear maturation of dromedary camel oocytes vitrified at the GV stage using different cryo-carriers and various vitrification solutions

**Fig 2:**
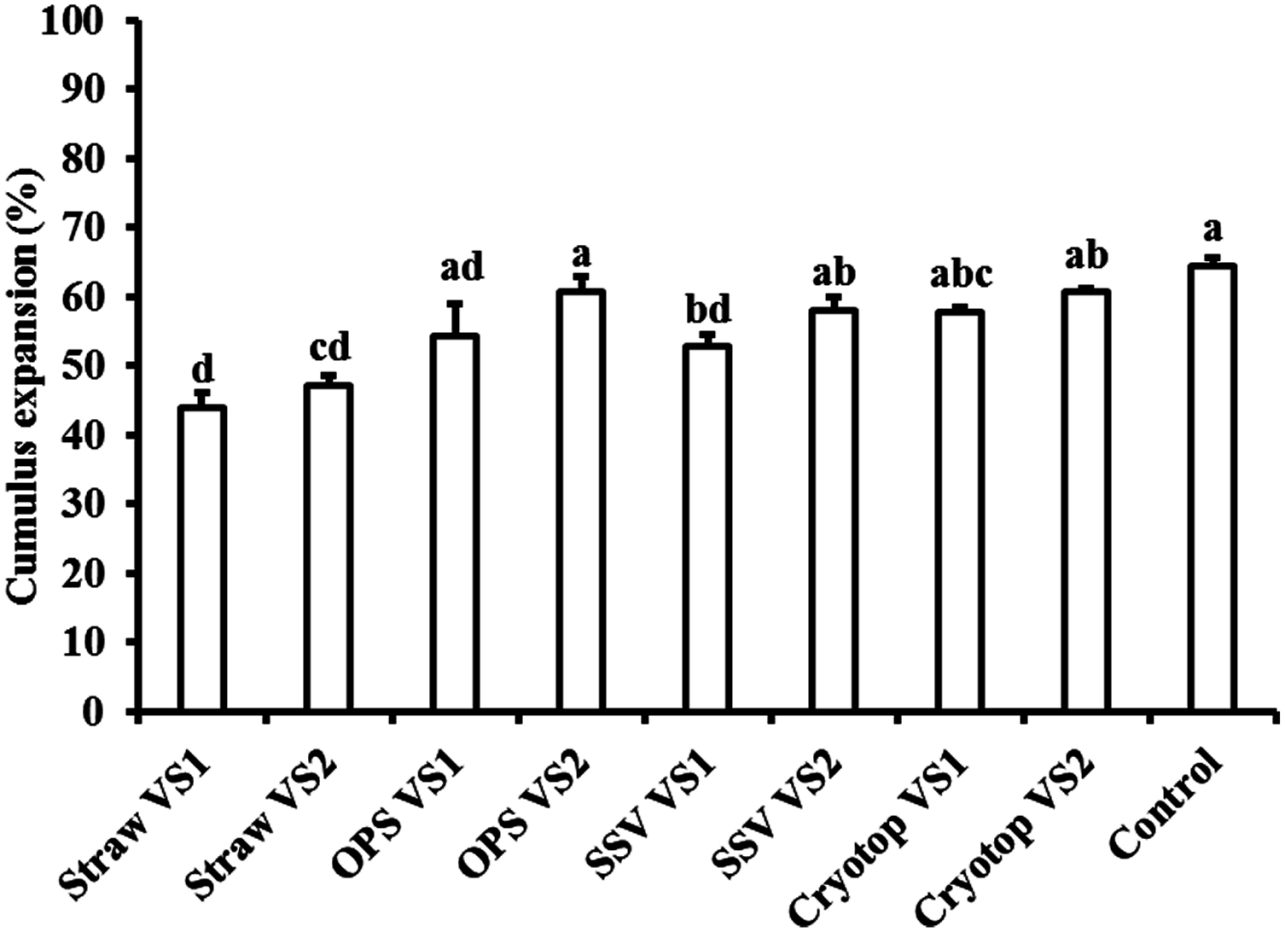
Cumulus cell expansion following in vitro maturation of dromedary camel oocytes vitrified at the germinal vesicle stage using different vitrification solutions and cryodevices. Percentages of IVM oocytes showing expanded cumulus cells after vitrification with VS1 (20% EG plus 20% DMSO) or VS2 (25% EG plus 25% DMSO) and with traditional straws, OPS (open pulled straws), SSV (solid surface vitrification) and Cryotop. Data are presented as the means ± S.E.M. Different small letters indicate significant differences at *P ≤* 0.05.

Following IVM for 30 h, the proportions of COCs with expanded cumulus cells were significantly lower (*P ≤* 0.05) in the groups vitrified by traditional straws (44.0% and 47.1% in VS1 and VS2, respectively) compared to those vitrified with Cryotop (57.8% and 60.6% in VS1 and VS2, respectively) and to those in the control group (64.5%). No significant differences were observed in cumulus cell expansion between Cryotop and OPS, with both vitrification solutions and the control group (Fig 2). Nuclear maturation rates (oocytes at MII-stage) were significantly higher (*P ≤* 0.05) in COCs vitrified by Cryotop using the VS2 solution (51.8%) compared to those vitrified using traditional straws (29.3% and 30.7% in VS1 and VS2, respectively) and OPS (37.9% and 44.9% in VS1 and VS2, respectively). Vitrification of COCs with traditional straws and OPS in both VS1 and VS2 or with SSV in VS1 significantly decreased (*P ≤* 0.05) the maturation rates compared with the controls (59.2%). No significant differences were recorded in nuclear maturation rates between Cryotop vitrified groups and the controls (Table 2).

**Table 2:** In vitro maturation of dromedary camel oocytes vitrified at the germinal vesicle stage using different vitrification solutions and various cryodevices.

Cryodevice	Cryoprotectant	Oocytes	MII oocytes n (mean% ± SEM)
**Straw**	VS1	58	17 (29.3 ± 0.7)^d^
	VS2	65	20 (30.7 ± 1.1)^cd^
**OPS**	VS1	69	26 (37.9 ± 0.7)^bc^
	VS2	72	33 (44.9 ± 4.1)^b^
**SSV**	VS1	63	29 (45.9 ± 3.0)^b^
	VS2	68	33 (47.6 ± 4.9)^ab^
**Cryotop**	VS1	55	27 (48.9 ± 1.1)^ab^
	VS2	60	31 (51.8 ± 1.0)^a^
**Control**		73	43 (59.2 ± 0.8)^a^

VS1—oocytes vitrified in a vitrification solution composed of 20% EG plus 20% DMSO.

VS2—oocytes vitrified in a vitrification solution composed of 25% EG plus 25% DMSO.

OPS—open pulled straws.

SSV—solid surface vitrification.

Values with different superscripts in the same column are significantly different at *P ≤* 0.05.

### Fertilization status after IVM and IVF of dromedary camel COCs vitrified at the GV stage

As shown in Table 3, vitrification of dromedary camel COCs using traditional straws significantly reduced (*P ≤* 0.05) fertilization rates (20.0% and 20.9% in VS1 and VS2, respectively) compared to those obtained in the other vitrified groups. Fertilization rates were significantly (*P ≤* 0.05) lower in the OPS vitrified groups (29.9% and 35.2% in VS1 and VS2, respectively) compared with those vitrified by Cryotop using VS1 solution and the controls. No significant differences were observed among the Cryotop-vitrified oocytes (37.0% and 39.2% in VS1 and VS2, respectively), the SSV-VS2 vitrified group (37.3%) and the control group (44.6%).

**Table 3:** Fertilization rates following IVM/IVF of dromedary camel oocytes vitrified at the germinal vesicle stage using different vitrification solutions and various cryodevices.

Cryodevice	Cryoprotectant	Oocytes	Fertilized oocytes n (mean% ± SEM)
**Straw**	VS1	55	11 (20.0 ± 1.3)^d^
	VS2	57	12 (20.9 ± 2.2)^d^
**OPS**	VS1	63	19 (29.9 ± 1.2)^c^
	VS2	68	24 (35.2 ± 2.1)^bc^
**SSV**	VS1	59	20 (33.9 ± 1.1)^abc^
	VS2	67	25 (37.3 ± 1.8)^abc^
**Cryotop**	VS1	60	22 (37.0 ± 1.5)^abc^
	VS2	64	25 (39.2 ± 0.8)^ab^
**Control**		74	33 (44.6 ± 0.1)^a^

VS1—oocytes vitrified in a vitrification solution composed of 20% EG plus 20% DMSO.

VS2—oocytes vitrified in a vitrification solution composed of 25% EG plus 25% DMSO.

OPS—open pulled straws.

SSV—solid surface vitrification.

Values with different superscripts in the same column are significantly different at *P ≤* 0.05.

### In vitro embryo development following IVM/IVF/IVC of dromedary camel COCs vitrified at the GV stage

Cleavage rates (48 hpi) were significantly lower (*P ≤* 0.05) in traditional straws (13.1% and 13.7% in VS1 and VS2, respectively), OPS (18.0% and 22.9% in VS1 and VS2, respectively) and SSV-VS1 (22.5%) vitrified oocytes than the control group (32.2%). Vitrification of COCs in traditional straws using VS1 or VS2 or with OPS using VS1 significantly (*P ≤* 0.05) reduced cleavage rates compared with those vitrified by the Cryotop (26.3% and 27.9% in VS1 and VS2, respectively). No significant differences were observed in cleavage rates between Cryotop-vitrified oocytes and the control group. Development to the morula stage (day 4 pi) did not significantly differ in COCs vitrified by OPS, SSV or Cryotop using both vitrification solutions (values ranged from 9.9% to 14.5%) and the control group (16.9%). Vitrification of COCs by using traditional straws significantly (*P ≤* 0.05) reduced morula development (5.7% and 6.9% in VS1 and VS2, respectively) compared to the control. Blastocyst development was significantly (*P ≤* 0.05) lower in COCs vitrified with traditional straws using VS1 or VS2 solutions (1.7%) compared with those vitrified by OPS in VS2 solution (6.1%), SSV (6.4% and 8.5% in VS1 and VS2 respectively), Cryotop (6.5% and 7.3% in VS1 and VS2, respectively) and the control (9.0%). Vitrification of COCs using OPS in a solution composed of 20% EG and 20% DMSO (VS1) resulted in significantly lower (*P ≤* 0.05) blastocyst formation rates (3.1%) compared to the control. No significant differences were identified in blastocyst rates between SSV, Cryotop-vitrified groups and the control (Table 4). The same trend was observed when blastocyst rates were calculated based on the number of cleaved embryos.

**Table 4:** Preimplantation embryo development following IVM/IVF/IVC of dromedary camel oocytes vitrified at the germinal vesicle stage using different combinations of cryoprotectants and cryodevices.

Cryodevice	Cryoprotectant	Oocytes	Cleavage n (mean% ± SEM)	Morula n (mean% ± SEM)	Blastocysts/oocyte n (mean% ± SEM)	Blastocysts/cleaved (%)
**Straw**	VS1	53	7 (13.1 ± 1.0)^d^	3 (5.7 ± 0.4)^c^	1 (1.7 ± 1.7)^c^	14.3^c^
	VS2	58	8 (13.7 ± 1.3)^d^	4 (6.9 ± 1.6)^bc^	1 (1.7 ± 1.7)^c^	12.5^c^
**OPS**	VS1	61	11 (18.0 ± 0.7)^c^	6 (9.9 ± 0.6)^ab^	2 (3.1 ± 1.6)^bc^	18.2^bc^
	VS2	65	15 (22.9 ± 1.8)^bc^	8 (12.2 ± 1.1)^ab^	4 (6.1 ± 1.3)^ab^	26.7^ab^
**SSV**	VS1	62	14 (22.5 ± 1.3)^bc^	9 (14.5 ± 0.2)^ab^	4 (6.4 ± 1.5)^ab^	28.6^ab^
	VS2	58	15 (25.7 ± 2.3)^abc^	8 (13.9 ± 2.0)^ab^	5 (8.5 ± 1.5)^a^	33.3^a^
**Cryotop**	VS1	60	16 (26.3 ± 2.9)^ab^	8 (13.2 ± 1.1)^ab^	4 (6.5 ± 1.3)^ab^	25.0^ab^
	VS2	68	19 (27.9 ± 0.9)^ab^	9 (13.4 ± 3.0)^ab^	5 (7.3 ± 1.3)^a^	26.3^a^
**Control**		65	21 (32.2 ± 1.4)^a^	11 (16.9 ± 3.0)^a^	6 (9.0 ± 2.3)^a^	28.6^a^

VS1—oocytes vitrified in a vitrification solution composed of 20% EG plus 20% DMSO.

VS2—oocytes vitrified in a vitrification solution composed of 25% EG plus 25% DMSO.

OPS—open pulled straws.

SSV—solid surface vitrification.

Values with different superscripts in the same column are significantly different at the *P ≤* 0.05 level.

## Discussion

In the present study, we demonstrate for the first time that dromedary camel oocytes vitrified at the GV-stage have the ability to be matured, fertilized and subsequently developed in vitro to produce blastocysts at frequencies similar to those seen using control (non-vitrified) oocytes. We also showed that both vitrification solutions and cryo-carriers affect the viability and developmental competence of vitrified/warmed dromedary camel oocytes. Furthermore, we revealed that both the survival and nuclear maturation of dromedary camel COCs were affected by the types and concentrations of the cryoprotectants used for vitrification with the lowest values seen in groups exposed to a combination of 25% EG plus 25% glycerol (VS3). Among the different vitrification solutions, the highest percentages of intact and mature oocytes were achieved in the VS2 group (Fig 1), indicating that a combination of 25% EG plus 25% DMSO is less toxic and may be used effectively during vitrification of dromedary camel GV-oocytes. Previous studies in sheep [28] showed that exposure of GV-oocytes to a vitrification solution composed of 25% EG and 25% DMSO for 60 sec significantly reduced in vitro maturation rates compared to those exposed to 20% EG and 20% DMSO (16.1% versus 50.0%). It has been shown that exposure of ovine GV-oocytes to a combination of 25% EG and 25% glycerol did not affect in vitro maturation rates compared to the control group (45.1% versus 61.4%) [28]; however, in our study, we found a severe reduction in maturation rates in the oocytes exposed to the same combinations of cryoprotectants. These results support the understanding of differential, species-specific sensitivity to cryoprotectants. The lower survival and maturation rates obtained in our studies after exposure of oocytes to VS3 could be due to the toxic effect of glycerol because it has low membrane permeability that causes osmotic damage to the cells [40]. A combination of 20% EG and 20% DMSO has been used effectively in oocyte vitrification in many animal species, including cattle [25], sheep [17, 19, 21, 28], and buffalo [24]. To optimize the protocol for the preservation of dromedary camel female germ lines, we evaluated the viability and development of dromedary camel GV-oocytes after vitrification and warming using four different cryodevices, straws, OPS, SSV, and Cryotop, and two vitrification solutions, VS1 and VS2.

Oocyte loss during vitrification and warming procedures has been reported in buffaloes [24], goats [41], sheep [19, 20], and mice [27]. This loss could occur as a consequence of osmotic injuries and/or due to sticking of oocytes to the pipette or cryo-carrier [27]. In our experiment, regardless of the vitrification solutions used, both SSV and Cryotop yielded the lowest recovery rates compared to those vitrified in straws using VS1 or VS2 or in OPS using VS2 (Table 1). The higher loss of oocytes in the SSV and Cryotop methods could be due to the small size of the droplets that have been utilized during vitrification. During warming, the oocytes within these droplets can float in the warming solutions, rendering their recovery more difficult. Furthermore, the chances of oocytes sticking to the inner wall of the handling pipette are high during SSV or Cryotop vitrification. Although recovery rates were the lowest in SSV and Cryotop, the percentage of intact oocytes was higher in those devices than with other carriers, indicating that both SSV and Cryotop could maintain the intactness of dromedary camel COCs after vitrification and warming. The high survival rates of dromedary camel oocytes reported here after SSV and Cryotop vitrification could be due to the high cooling and warming rates obtained by these two methods. Similar to our results, previous studies in cattle reported high survival rates (over 90%) of vitrified/warmed MII-oocytes by using SSV and Cryotop [42]. Recent studies showed that vitrification of alpaca in vitro matured oocytes using SSV- and EG-based vitrification solutions significantly decreased oocyte viability compared to non-vitrified controls (76.4% versus 97.3%) [43].

In vitro maturation, fertilization and development of oocytes after cryopreservation are imperative requirements in the success of oocyte cryopreservation. The effects of vitrification and warming of COCs at the GV-stage on cumulus cell expansion after IVM have been reported across different species, and the results are still controversial and dependent on the individual species. For example, recent studies in buffalo showed that the percentages of oocytes with expanded cumulus cells after IVM did not significantly differ between the vitrified and the control groups (74.9% versus 88.1%) [24]. On the other hand, vitrification of ovine GV-oocytes using SSV or cryoloop significantly decreased the proportions of oocytes with expanded cumulus cells following IVM (41.3% and 70.4%, respectively) compared to the control group (95.2%) [19, 20]. In the current study, we found that vitrification of dromedary camel COCs with OPS or Cryotop did not adversely affect cumulus cell expansion after IVM; however, vitrification with traditional straws reduced the percentage of oocytes with expanded cumulus cells (Fig 2). These findings confirm that the quality of the oocytes and their response to vitrification and warming differ among species. Interestingly, in the present study, we showed that vitrification of COCs with Cryotop or with SSV in VS2 did not negatively impact nuclear maturation rates after IVM; however, straws and OPS vitrification did. These results confirm that the vitrification method and cryoprotectants affect the quality of oocytes after vitrification and warming in dromedary camels. A number of previous studies have also reported lower maturation rates following cryopreservation of GV–oocytes in multiple species, including cattle [44], sheep [20], horses [30], buffalos [24], cats [45], mice [16], and humans [46, 47]. Recently, it has been shown that dromedary camel GV-oocytes, obtained from vitrified/warmed ovarian fragments using SSV, can resume meiosis, although the frequencies were significantly lower than those obtained from control non-vitrified oocytes (25.9% versus 56.6%) [48]. The lower maturation rates after vitrification and warming of GV-oocytes can be ascribed to the damage to certain organelles in the cytoplasm that are pivotal for oocyte maturation [16, 19]. Interestingly, in the present study, the maturation rates of oocytes in the control group (59.2%) were comparable to most of the previous results following IVM of dromedary oocytes, namely, 54.9% [49], 63.0% [50], and 51.4% [35]. However, they were still lower than the 81.4% reported by Wani and Wernery [51]. Various factors, such as quality of oocytes, season effect, the duration of oocyte maturation, maturation medium and supplements, and culture conditions, could contribute to the differences between our results and the findings of Wani and Wernery [51].

Oocyte vitrification has been reported to decrease in vitro fertilization rates in different species such as cattle [44], sheep [20], and pigs [52]. The authors ascribed these differences in fertilization rates to many factors including the premature release of cortical granules, zona hardening and/or parthenogenetic activation of cryopreserved oocytes [11]. We showed that vitrification of COCs with SSV or Cryotop did not cause any defects in fertilization, indicating that vitrification of dromedary camel COCs using SSV or Cryotop, in either VS1 or VS2, may be an alternative to circumvent the problems associated with fertilization defects during cooling and warming procedures. Similar to our results, previous studies reported that the vitrification of bovine IVM oocytes using SSV or Cryotop did not significantly affect fertilization rates compared to controls (36.0%, 34.0% and 40.0%, respectively) [42]. Interestingly, in our experiments, the percentage of fertilized oocytes (44.6%) in the control groups is higher than those reported in llama (29.2%) [53]. However, our values are lower than those reported in the same species after IVF of IVM oocytes, 52.0% [54], 68.0% [37] and 58.97% [35]. These variations in the results may be attributed to several factors, such as the quality of sperm, age of the animal, sperm concentration used for IVF, duration of IVF, fertilization medium, seasonality effects, the use of the animal in natural or artificial breeding, methods of semen preparation, and the quality and IVM conditions of oocytes.

Perturbations resulting from oocyte cryodamage may also appear during cleavage and early embryo development [55]. Many studies in different species reported lower cleavage and blastocyst rates following IVF of vitrified/warmed oocytes compared with the controls [15, 17–20]. Interestingly, we found that vitrification of dromedary camel COCs at the GV-stages using SSV or Cryotop in VS2 solution (25% EG plus 25% DMSO) did not adversely influence cleavage and blastocyst development after IVM, IVF and embryo culture; however, these parameters were dramatically reduced in straw-vitrified oocytes. The present findings confirm that the developmental competence of vitrified/warmed dromedary oocytes depends on the vitrification method and cryoprotectants used. The lack of studies regarding cryopreservation of dromedary camel oocytes hampers comparison of our results because most of the available reports come from domestic ruminants. However, previous studies on alpacas showed that vitrification of in vitro matured oocytes in a vitrification solution composed of 35% EG significantly reduced blastocyst development after parthenogenetic activation compared to the control (7.5% vs. 12.1%, respectively) and this percentage decreased by increasing the exposure time to the vitrification solution up to 45 sec [43]. The proportions of blastocysts reported in the current study from the control oocytes (28.6%, based on the numbers of cleaved embryos) were higher than most reported results after IVM/IVF, such as llama oocytes (4.7%) [53] and dromedary oocytes (16%) [50]. However, the values were lower than one report (35%) [56] following IVF of dromedary camel oocytes. The difference may reveal variations in maturation conditions and the nature of the spermatozoa used in IVF (freshly ejaculated vs. epididymal).

Taken together, our results demonstrate that SSV and Cryotop vitrification methods are equally effective for the cryopreservation of dromedary camel COCs at the GV stage; both methods resulted in high survival, maturation, fertilization, cleavage and preimplantation embryo development. Indeed, both of the techniques can be recommended for the cryopreservation of dromedary camel oocytes. The choice between the two methods may depend on the advantages and availability of each method, as well as on the number of oocytes undergoing cryopreservation. Although SSV is a cheaper method by which large numbers of oocytes can be preserved in a short time, it is hard to control the size of the droplet, and thus, the chances for losing the oocytes are high. However, with Cryotop vitrification, the volume of the solution and oocyte number can be controlled. However, the number of oocytes that can be preserved with each Cryotop is limited, and it takes a long time to preserve large numbers of oocytes. In conclusion, we show that both the vitrification solution and cryodevice affect the survival and developmental competence of vitrified/warmed dromedary camel oocytes. We report for the first time that dromedary camel oocytes vitrified at the germinal vesicle stage have the ability to be matured, fertilized and subsequently cultured in vitro to produce blastocysts at frequencies comparable to those obtained using fresh oocytes. Both SSV and Cryotop as cryo-carriers in combination with VS2 (25% EG plus 25% DMSO) could be used effectively for the vitrification of dromedary camel GV-oocytes. However, further studies are warranted to assess the ability of in vitro-produced blastocysts to develop in vivo, establish pregnancy and produce live offspring when transferred to surrogate mothers.
